# A novel endoscopic suturing device: comparison with endoclips and hand-sewn techniques for gastrostomy closure in an ex vivo porcine model

**DOI:** 10.1016/j.igie.2024.10.004

**Published:** 2024-10-22

**Authors:** Jun Hee Lee, Ji Yoon Kim, Taebin Kwon, Hyuk Soon Choi, Bora Keum, Hoon Jai Chun, Daehie Hong, Hyunsoo Chung

**Affiliations:** 1 Department of Internal Medicine and Liver Research Institute, Seoul National University College of Medicine, Seoul, Republic of Korea; 2 EndoRobotics Co, Ltd, Seoul, Republic of Korea; 3 Department of Internal Medicine, Korea University College of Medicine, Seoul, Republic of Korea; 4 Department of Medical Device Development, Seoul National University Graduate School, Seoul, Republic of Korea

As endoscopic interventions increasingly replace a significant portion of surgical treatments, demand is growing for full-thickness suturing, particularly in gastroenterology, for the management of perforations, fistulas, and endoscopic gastroplication.^1^ Endoclips do not allow full-thickness closure in most cases, especially in the stomach. OverStitch (Appollo Endosurgery, Austin, Tex, USA) is an endoscopic suturing system currently approved for clinical use that enables full-thickness suturing. However, it is challenging to rotate the needle position or change the stitch direction (eg, from left to right or right to left) during the procedure.^2^, ^3^, ^4^

We have developed a new endoscopic full-thickness suture device that addresses the shortcomings of existing devices. It offers bidirectional suturing and precise control of suture direction and position, reducing manual effort and physical strain on the endoscopist. The aim of the current study was to compare the novel endoscopic suturing device with hand-sewn techniques and endoclipping for gastrotomy closure in an ex vivo porcine model.

## Methods

A leak test was performed to evaluate the strength of closures using 3 methods: endoscopic suturing, endoclip placement, and hand-sewn suturing. A total of 30 ex vivo porcine stomachs were used, with 10 stomachs randomly assigned to each group. Each group attempted closure of a 2-cm-long full-thickness linear perforation created on the lesser curvature side of the stomach body with a standardized device. After sealing open areas with clamps and zip ties, the stomach sample was placed in a water tank, and air was injected using an air pump (KOL-A20, KOLAVO, Seoul, Republic of Korea). The pressure at which leakage occurred was then measured. All procedures were conducted by a board-certified GI endoscopist.

For the endoscopic suturing group, closures were made using an endoscope and suturing device with 4 to 5 simple continuous stitches (average, 4.2) using a stainless steel-tipped needle (V-Loc 180 reload 2-0; Covidien, Minneapolis, Minn, USA) and endoscopic scissors (Ensizor; Slater Endoscopy, Miramar, Fla, USA). In the clip closure group, 4 to 6 endoscopic clips (average, 5) were used with the EZ Clip and clipping applicator (Olympus, Tokyo, Japan). For the hand-sewn group, suturing was performed on the stomach’s outer surface with 4 simple continuous stitches using the same needle as the endoscopic group.

### Endoscopic methods

**Figure 1:**
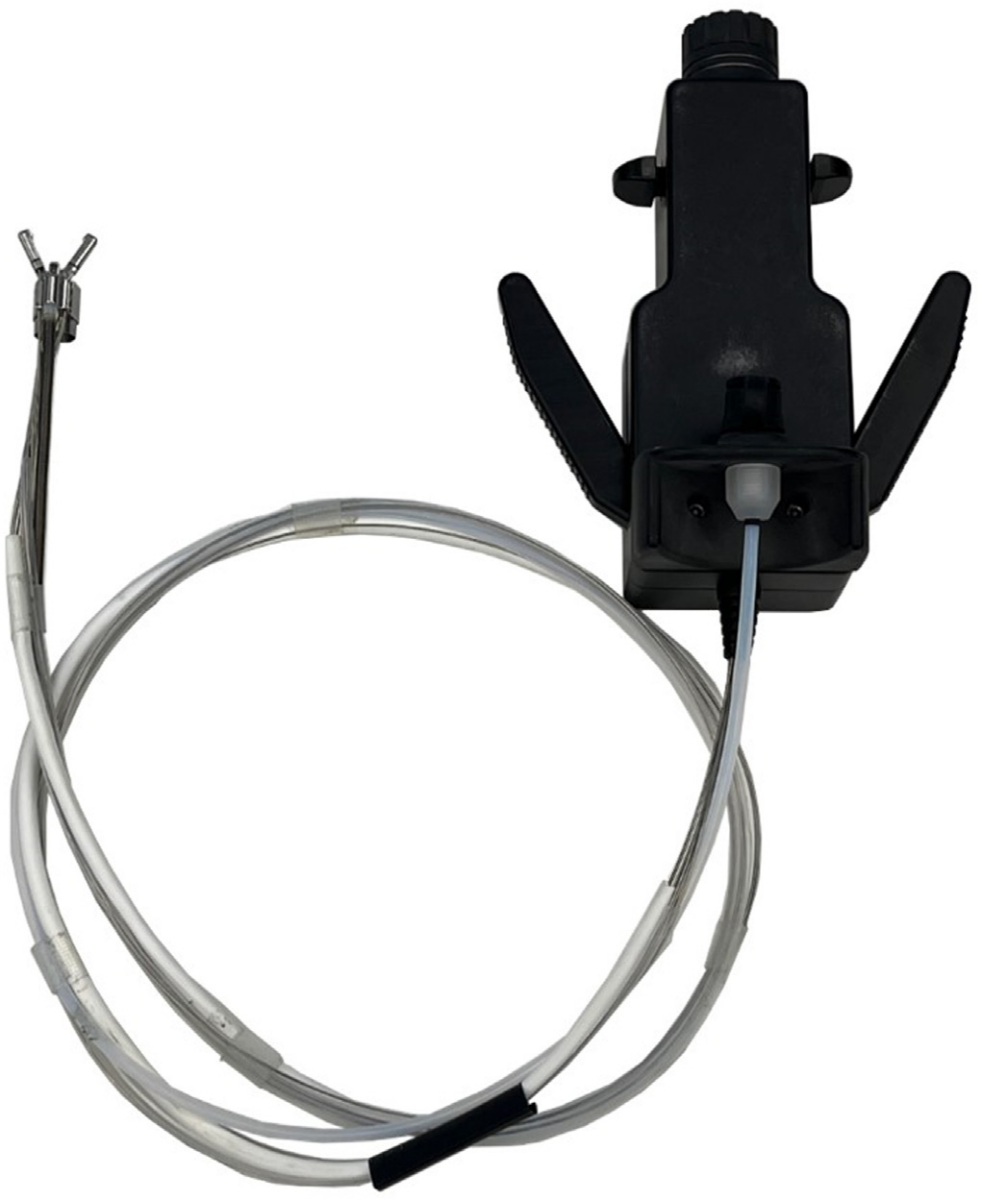
Image of a novel endoscopic suturing device.

The suturing device consists of an end effector with 2 jaws, a bundle of cables approximately 1 m long, and a manual controller (Fig. 1). The end effector is mounted at the tip of a gastroscope using the over-the-scope method. The end effector performs suturing by exchanging a needle between its 2 jaws, with the ability to adjust suturing direction and position through a rolling motion (Video 1, available online at www.igiejournal.org).

The needle (V-Loc 180 reload 2-0; Covidien) used in the suturing device is a commercial product with a stainless steel tip specifically designed to eliminate the need for an additional cinching process. The manual controller attaches to the boot portion of the endoscope and operates the end effector, which consists of 2 jaws, an additional channel, a base, and a link. The end effector can perform 3 motions: (1) jaw opening and closing; (2) needle holding left and right; and (3) rolling motion clockwise and counterclockwise.

The manual controller features a holder at the back for easy attachment to the boot, adjustable via a screw. It includes handles on each side that, when pulled, close the end effector. Levers located above the handles allow the needle to pass from side to side, and a round knob at the top enables rotation of the end effector both clockwise and counterclockwise (Supplementary Fig. 1, available online at www.igiejournal.org).

### Statistical analysis

For the leak test, the Kruskal-Wallis test was used because the endoclip group did not satisfy the normality test, whereas the suturing device and hand-sewn groups did. For the procedure time analysis, a Student *t* test was performed. A significance level of <.05 was considered statistically significant. All statistical analyses were conducted by using IBM SPSS Statistics 27.0 (IBM SPSS Statistics, IBM Corporation, Armonk, NY, USA).

## Results

**Figure 2:**
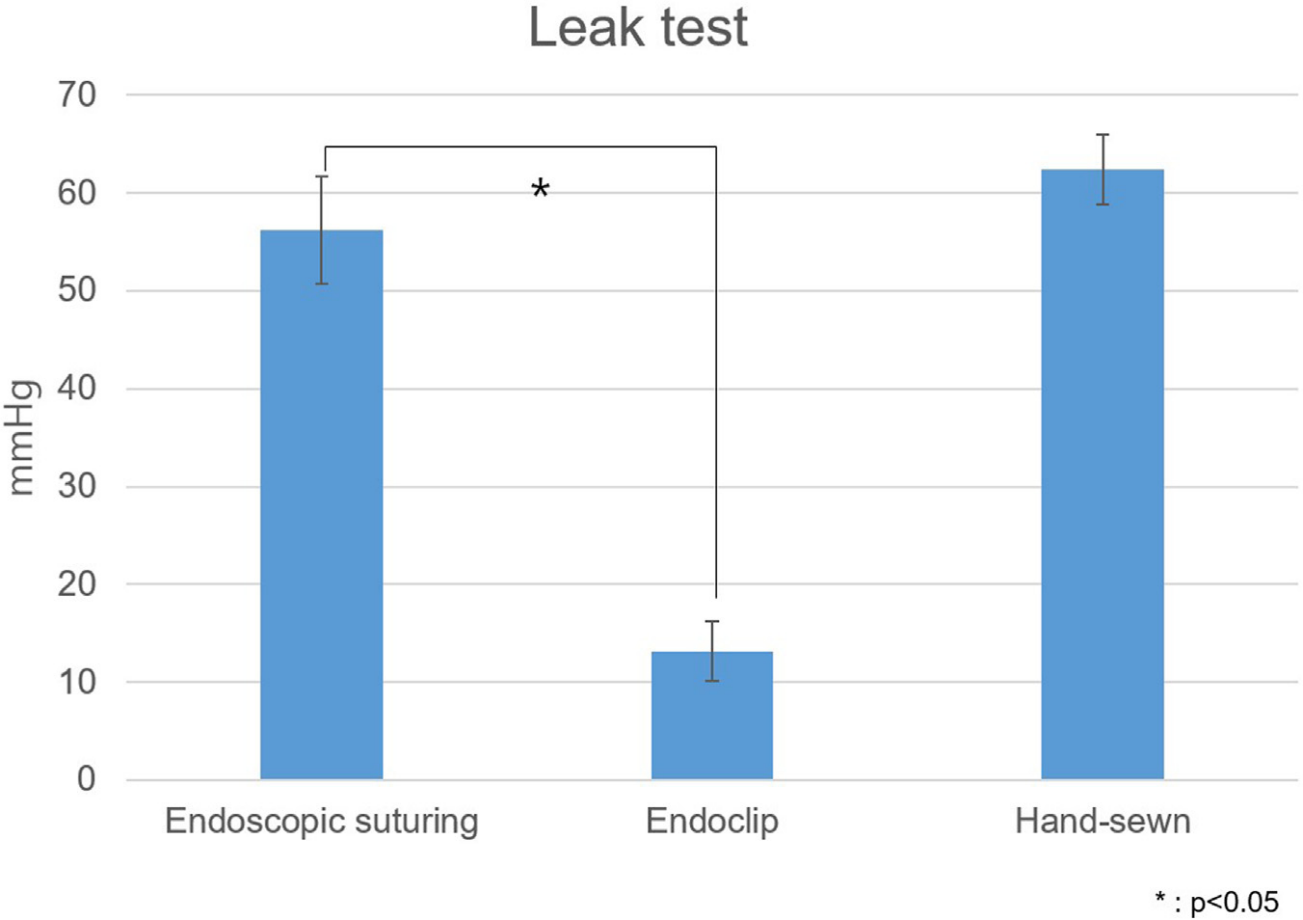
Leak pressure analysis comparing endoscopic suturing, endoclip, and hand-sewn suturing.

**Figure 3:**
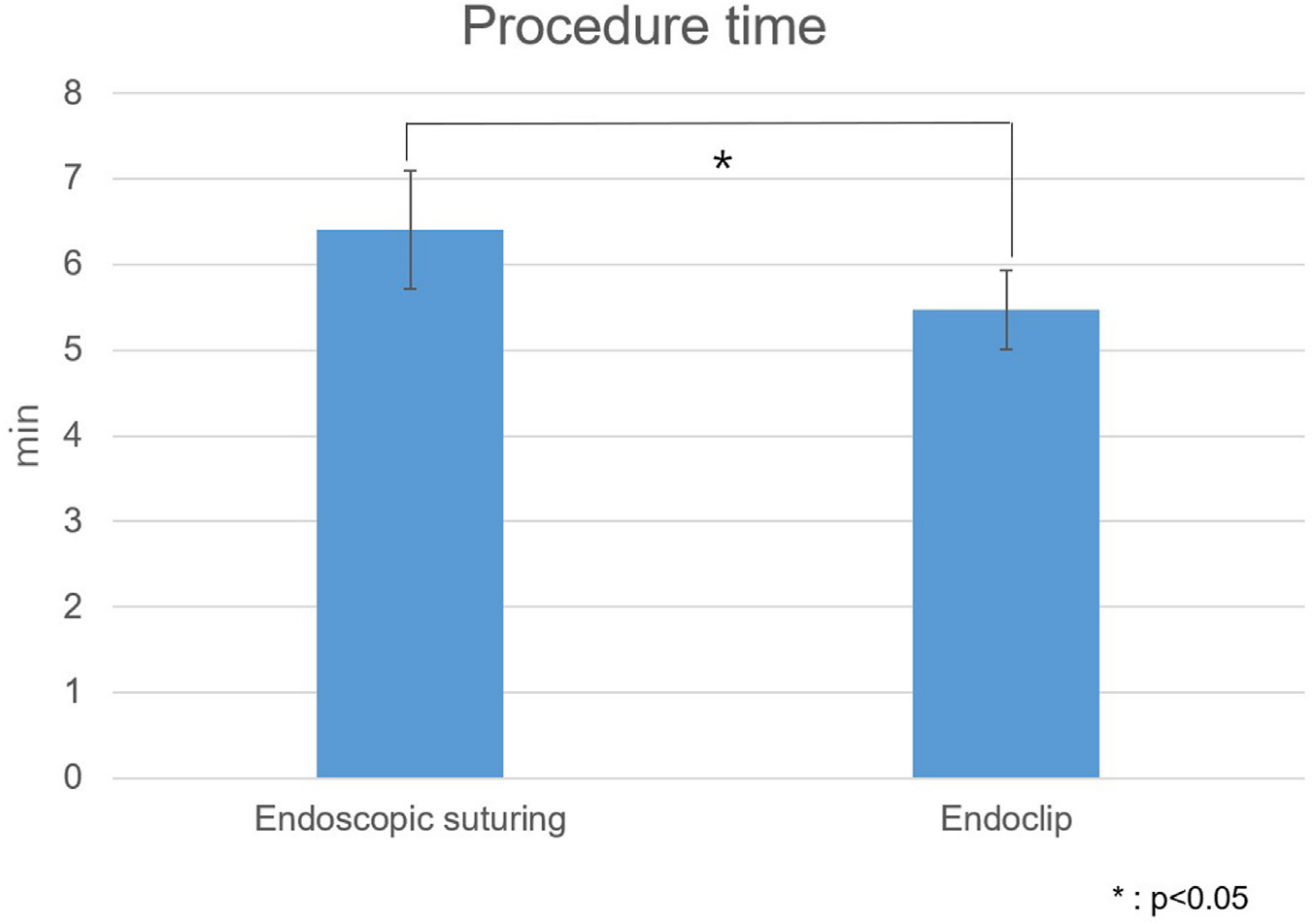
Procedure time analysis comparing endoscopic suturing and endoclips.

The leak pressure in the endoscopic suturing group was 56.2 ± 17.4 mm Hg, significantly higher than the 13.2 ± 9.5 mm Hg observed in the endoclip group (*P* < .001). The leak pressure in the hand-sewn group was 62.4 ± 11.1 mm Hg, which did not differ significantly from that in the endoscopic suturing group (*P* > .05) (Fig. 2). For the comparison of procedure time, hand-sewn suture cases were excluded due to their inconsistent surgical environment, and comparisons were made between cases using the device and endoclip. The procedure time for the endoscopic suturing group was 6.4 ± 2.2 minutes, which differed significantly from the 5.5 ± 1.5 minutes observed for the endoclip group (*P* < .05) (Fig. 3).

## Conclusions

In this ex vivo porcine stomach model, we showed that the leak pressure of the novel endoscopic full-thickness suture was significantly higher than that of endoclip closure, with no significant difference observed compared with the hand-sewn technique. However, the analysis of procedure time indicated that the endoscopic suturing group required more time than the endoclip group. This result is partly unavoidable, as endoscopic suturing involves more detailed steps and, being an innovative procedure, comes with a learning curve. We anticipate that as endoscopists gain more experience with the device, the procedure time can be reduced. To evaluate efficacy and safety, further comparative studies with existing endoscopic suturing devices are necessary using an in vivo porcine model.

## Disclosure

All authors disclosed no financial relationships.

## Supplementary data

Supplementary Figure 1 Manipulation of the manual controller. (**A**) Front and back images of a manual controller attached to the boot portion of an endoscope. (**B**) Jaw opening and closing by manipulating the handles of the manual controller. (**C**) Needle holding and passing by manipulating the levers placed above the handles after closing the jaw. (**D**) Rotating the end effector by turning the round knob at the top of the manual controller clockwise and counterclockwise.

Video 1 . A novel endoscopic suturing device: comparison with hand-sewn techniques for gastrostomy closure in ex vivo porcine model.
