# Supplementary figures and images for: A novel endoscopic suturing device: comparison with endoclips and hand-sewn techniques for gastrostomy closure in an ex vivo porcine model
Source: IGIE. 2024 Oct 22;3(4):487–9. doi: 10.1016/j.igie.2024.10.004 (PMC12850846; doi:10.1016/j.igie.2024.10.004)

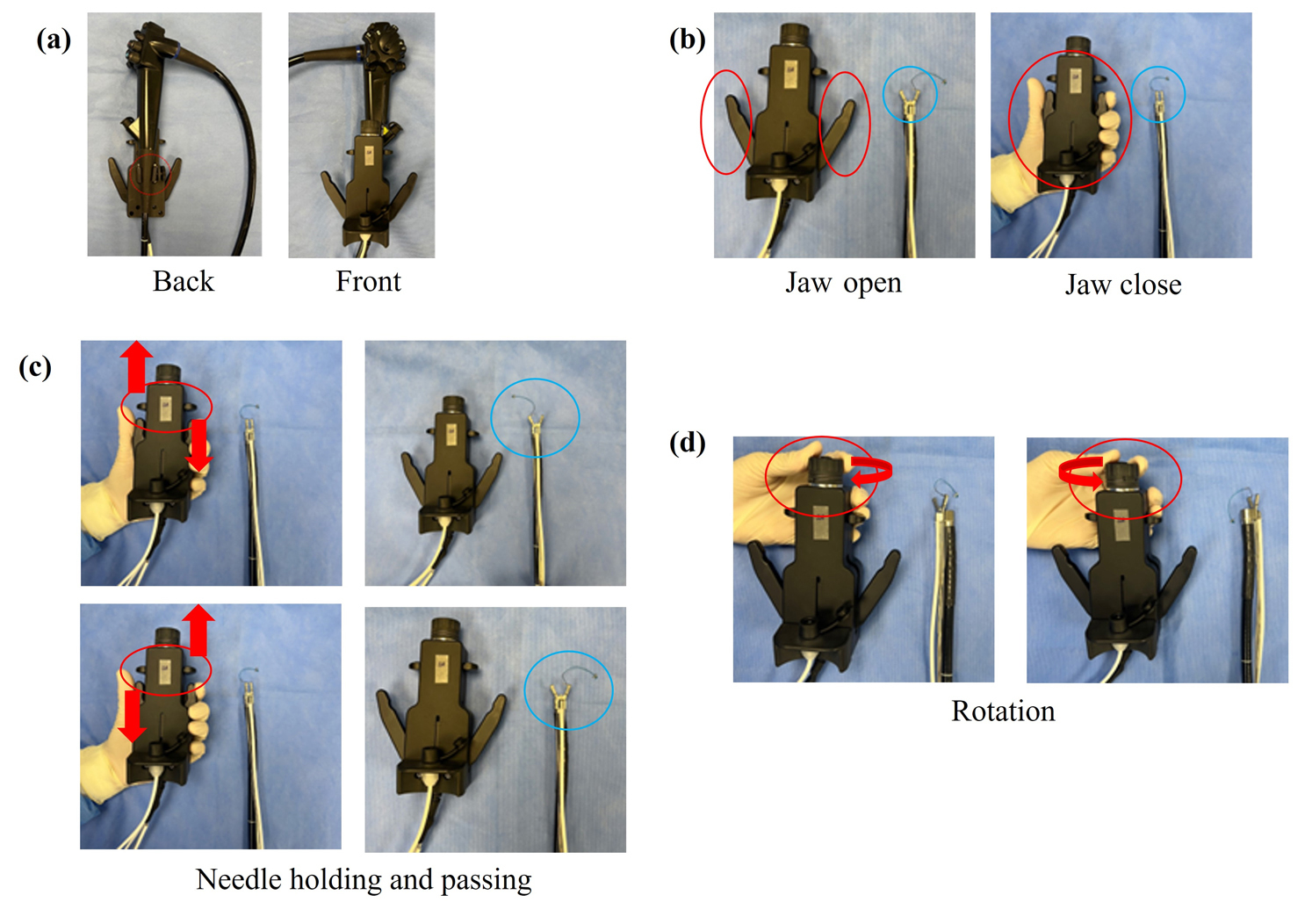

Supplement: Supplementary Figure 1 — Manipulation of the manual controller. (A) Front and back images of a manual controller attached to the boot portion of an endoscope. (B) Jaw opening and closing by manipulating the handles of the manual controller. (C) Needle holding and passing by manipulating the levers placed above the handles after closing the jaw. (D) Rotating the end effector by turning the round knob at the top of the manual controller clockwise and counterclockwise. [file figs1.jpg]
